# Supplementary material for: Microtubules as a versatile reference standard for expansion microscopy
Source: Commun Biol. 2025 Mar 26;8:499. doi: 10.1038/s42003-025-07967-3 (PMC11947214; doi:10.1038/s42003-025-07967-3)
Supplement: Supplementary file 2 — Supplementary Information [file 42003_2025_7967_MOESM2_ESM.pdf]

Microtubules as a versatile reference standard for expansion microscopy

Rajdeep Chowdhury[1,2*], Tiago Mimoso[3*], Abed Alrahman Chouaib[4], Nikolaos Mougios[1,5], Donatus Krah[1], Felipe Opazo[1,5,6], Sarah Köster[3,7§], Silvio O. Rizzoli[1,5,7§], Ali H. Shaib[1§]

[1]Department of Neuro- and Sensory Physiology, University Medical Center Göttingen, Göttingen, Germany.
[2]Department of Chemistry, GITAM School of Science, GITAM, Hyderabad 502329, Telangana, India.
[3]Institute for X-Ray Physics, University of Göttingen, Göttingen, Germany.
[4]Department of Cellular Neurophysiology, Center for Integrative Physiology and Molecular Medicine (CIPMM), Saarland University, Homburg, Germany.
[5]Center for Biostructural Imaging of Neurodegeneration, University Medical Center Göttingen, Göttingen, Germany.
[6]NanoTag Biotechnologies GmbH, Rudolf-Wissell-Straße 28a, 37079 Göttingen, Germany.
[7]Cluster of Excellence "Multiscale Bioimaging: from Molecular Machines to Networks of Excitable Cells" (MBExC), University of Göttingen, Göttingen, Germany.

*equal first authors, alphabetically.

§corresponding authors, alphabetically.
Corresponding authors' email addresses: Sarah Köster (sarah.koester@uni-goettingen.de), Silvio O. Rizzoli (srizzol@gwdg.de) and Ali H. Shaib (ali.shaib@med.uni-goettingen.de).

Supplementary Information

Supplementary Figures 1/9

Supplementary References 9/9

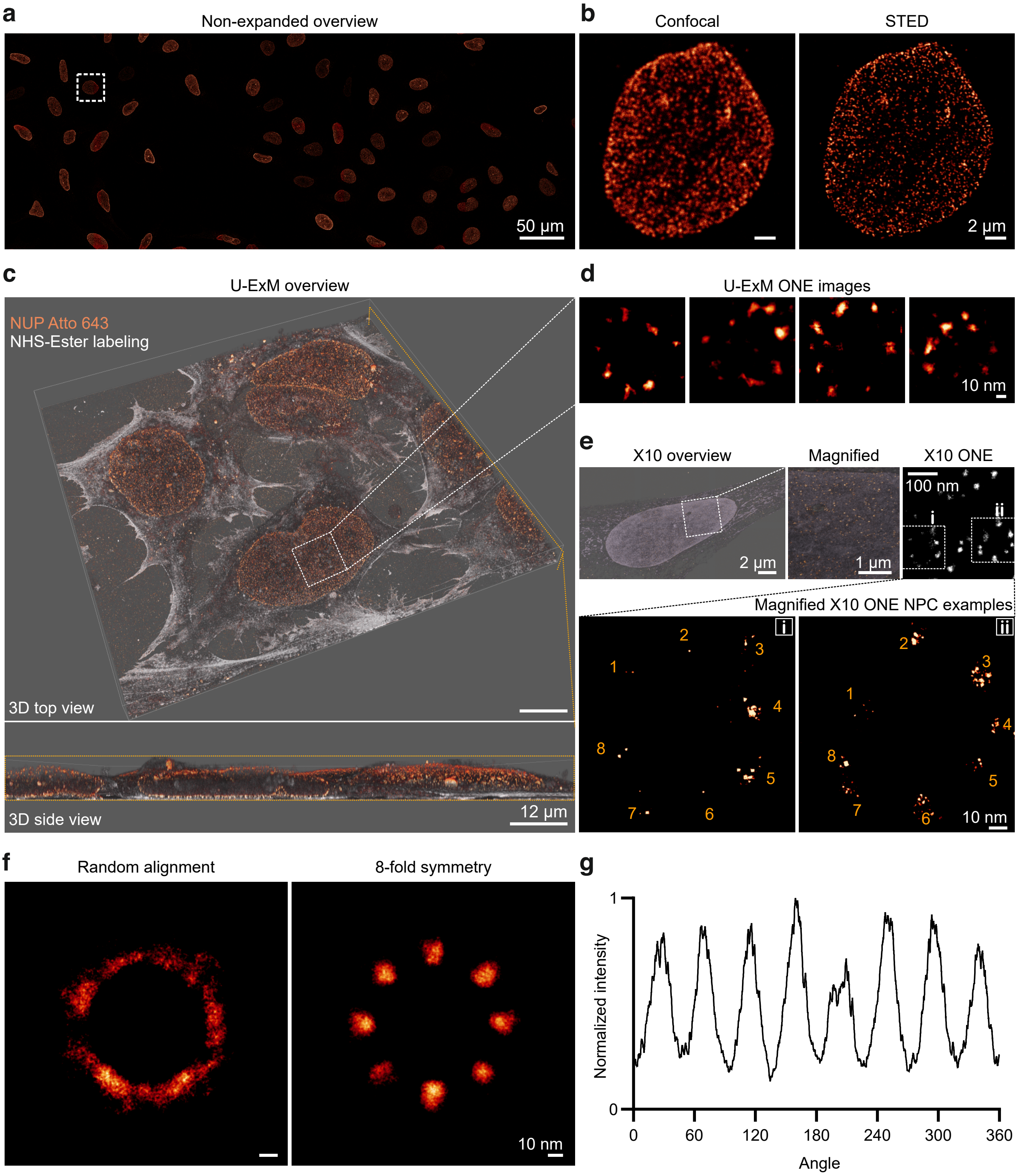

Supplementary Fig. 1. ExM imaging of nuclear pore complexes. a, **b**, non-expanded NPC overview and a representative magnified nucleus shown in confocal and 2D-STED acquisitions. **c**, A representative 3D-overview of HEK cells that were expanded according to the U-ExM protocol described in Gambarotto *et al.*, 2021[1] and were post-expansion labelled against NUP98 and NUP205 with primary rabbit antibodies coupled to secondary anti-rabbit nanobodies carrying STAR635P fluophores. The gels were then labelled with NHS-ester fluorescein, to show the general cellular morphology. **d**, U-ExM ONE microscopy images of NPCs from the designated area shown in (**c**). **e**, Similar overview but for X10 expansion, reaching an expansion factor of 8.5. A similar labeling procedure was performed, prior to expansion. The two lower panels show two representative NPCs from the upper image. **f**, A random averaged alignment of NPCs (left panel), followed by an 8-fold symmetry average alignment (right panel). **g**, A circle profile that shows the distribution of the NUPs in the right panel of (**f**). The averaging procedure of 566 NPC was performed as described in Aktalay *et al.*, 2023[2], for MINFLUX imaging of NPCs. The resulting averaged 566 NPC data, from two independent experiments, are in very good agreement with the MINFLUX results, especially considering the signal blurring induced by the use of polyclonal antibodies in our experiments, rather than a NUP96-HaloTags in MINFLUX.

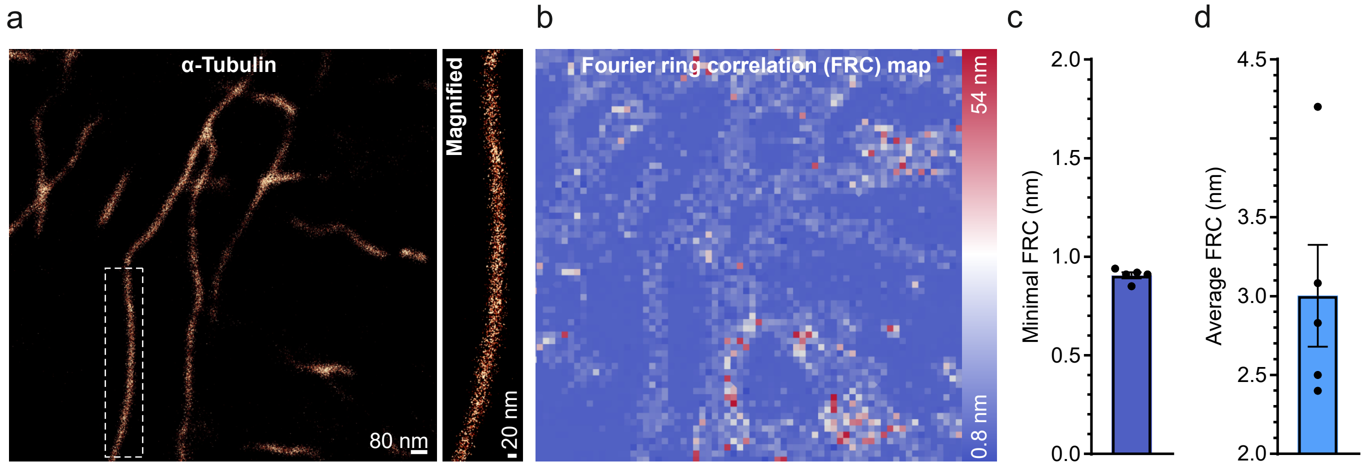

Supplementary Fig. 2. Fourier ring correlation analysis of tubulin immunostainings. Cells were labeled with primary antibodies against alpha-tubulin, followed by secondary nanobody labeling. **a**, ONE overview image followed by a magnified segment. **b**, Fourier ring correlation map. **c** & **d**, the graphs show minimal and average FRC in nm with their SEM, N = 5 measurements from 2 independent experiments.

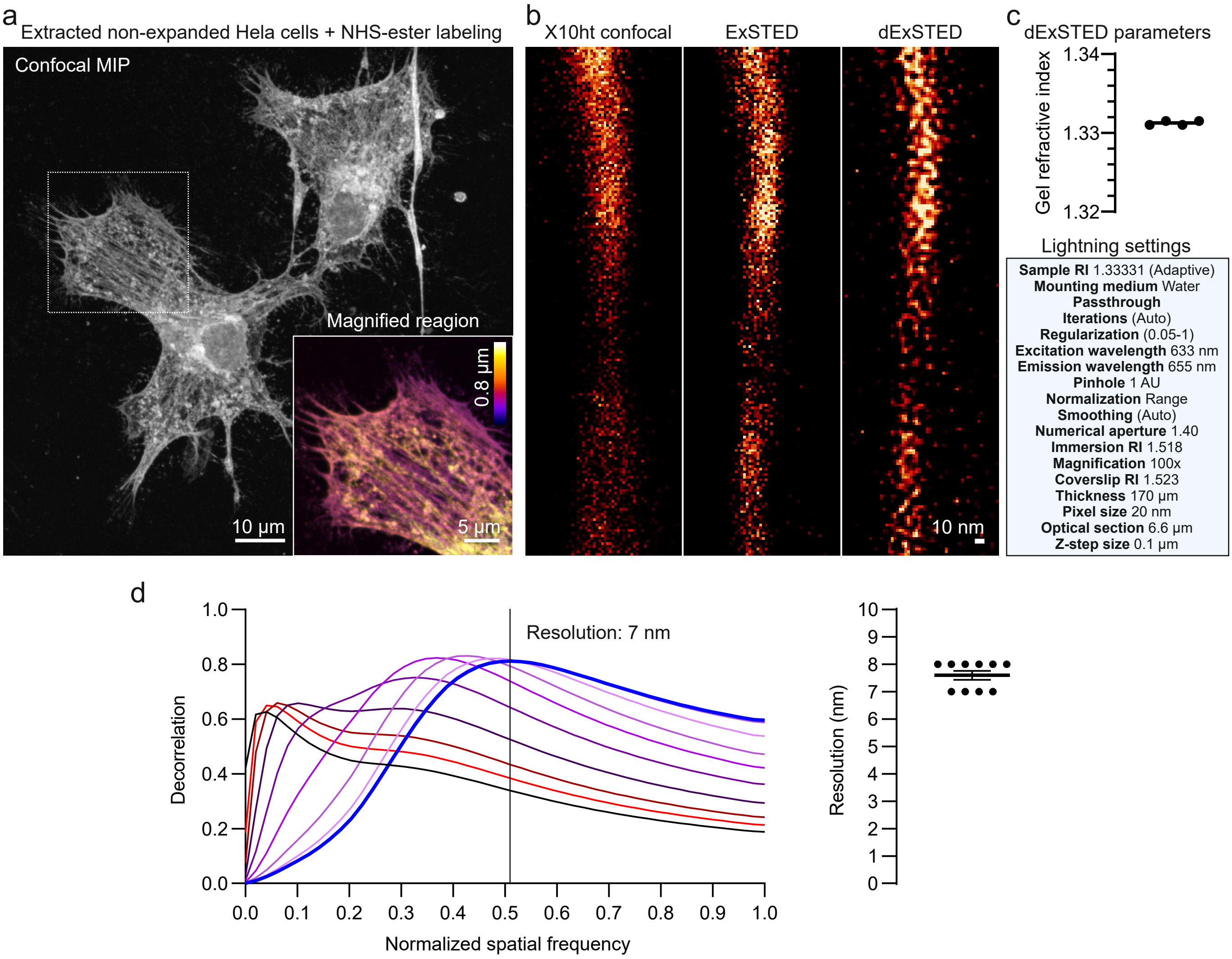

Supplementary Fig. 3. Deconvolved ExSTED parameters. a, An overview of extracted non-expanded HeLa cells labeled with NHS-ester fluorescein. **b**, An exemplary region of extracted and expanded HeLa cells featuring a tubulin segment imaged with X10ht confocal, ExSTED and dExSTED. **c**, A box plot graph depicting the average measured gel-glass refractive index (RI) measured from four gels of 4 independent experiments, using Abbe-Refractometer AR4 (Kruss Optronic GmbH, Germany). The obtained RI was then used for the deconvolution settings, which was carried out via Lightning options in LAS-X Leica software on a STELLARIS 8 microscope. The table lists the used parameters. **d**, The first panel shows a decorrelation analysis[3] plot of all decorrelation functions computed for estimating image resolution. The second box plot shows the individual values calculated from 10 dExSTED images acquired from 10 different cells from two independent experiments.

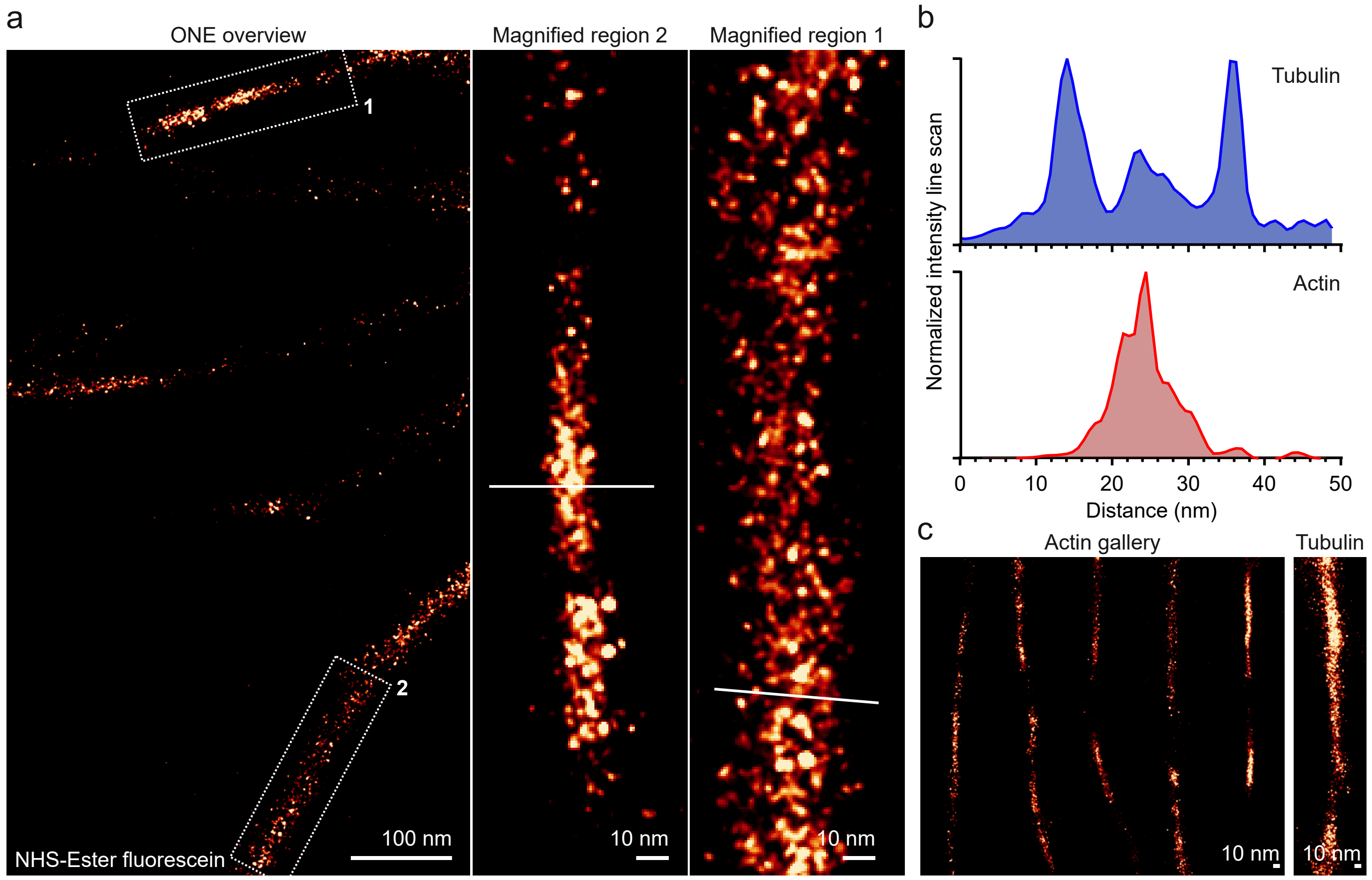

Supplementary Fig. 4. Cytoskeletal elements from extracted cells can be identified based on width measurements. a, The first panel shows an ONE overview image where extracted cytoskeletal elements of varying widths are typically observed in at least 4 independent experiments. The second two panels show two magnified regions, with cross sections that fit the profiles of actin and tubulin structures, as shown in the adjacent graph in (**b**). **c**, An exemplary gallery of actin images, next to an exemplary tubulin segment, for comparison.

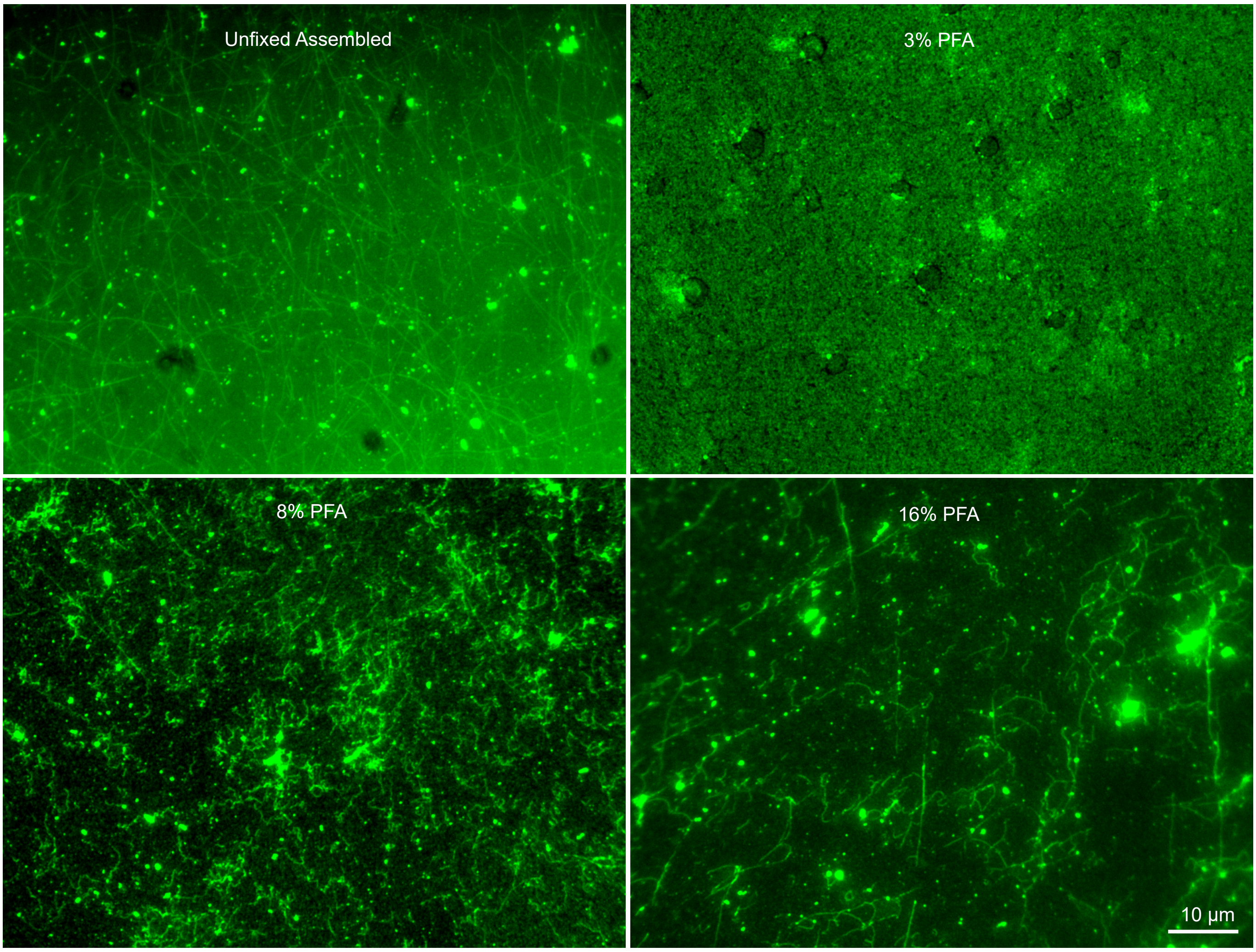

Supplementary Fig. 5. PFA fixation of microtubules. This type of fixation provided sub-optimal preservation of structure, although it does not interfere with anchoring, as up to 8% PFA allows ONE imaging. Microtubules are synthesized *in vitro*, stabilized with taxol (labelled with atto488), fixed with different concentrations of PFA, attached as a monolayer onto the coverslip, and imaged using the fluorescence of atto488 under an Olympus IX71 microscope. (Left, top) Not fixed, long filaments were clearly visible. (Right, top) Fixed with 3% PFA, all the microtubules were disassembled, presumably into tubulin dimers. (Left, bottom) Fixed with 8% PFA, most of the long filaments were broken into shorter ones, which is the only condition which worked with ExM protocols and ONE microscopy. (Right, bottom) Fixed with 16% PFA, a greater number of long filaments were preserved compared to 8% PFA, although very long ones were still broken.

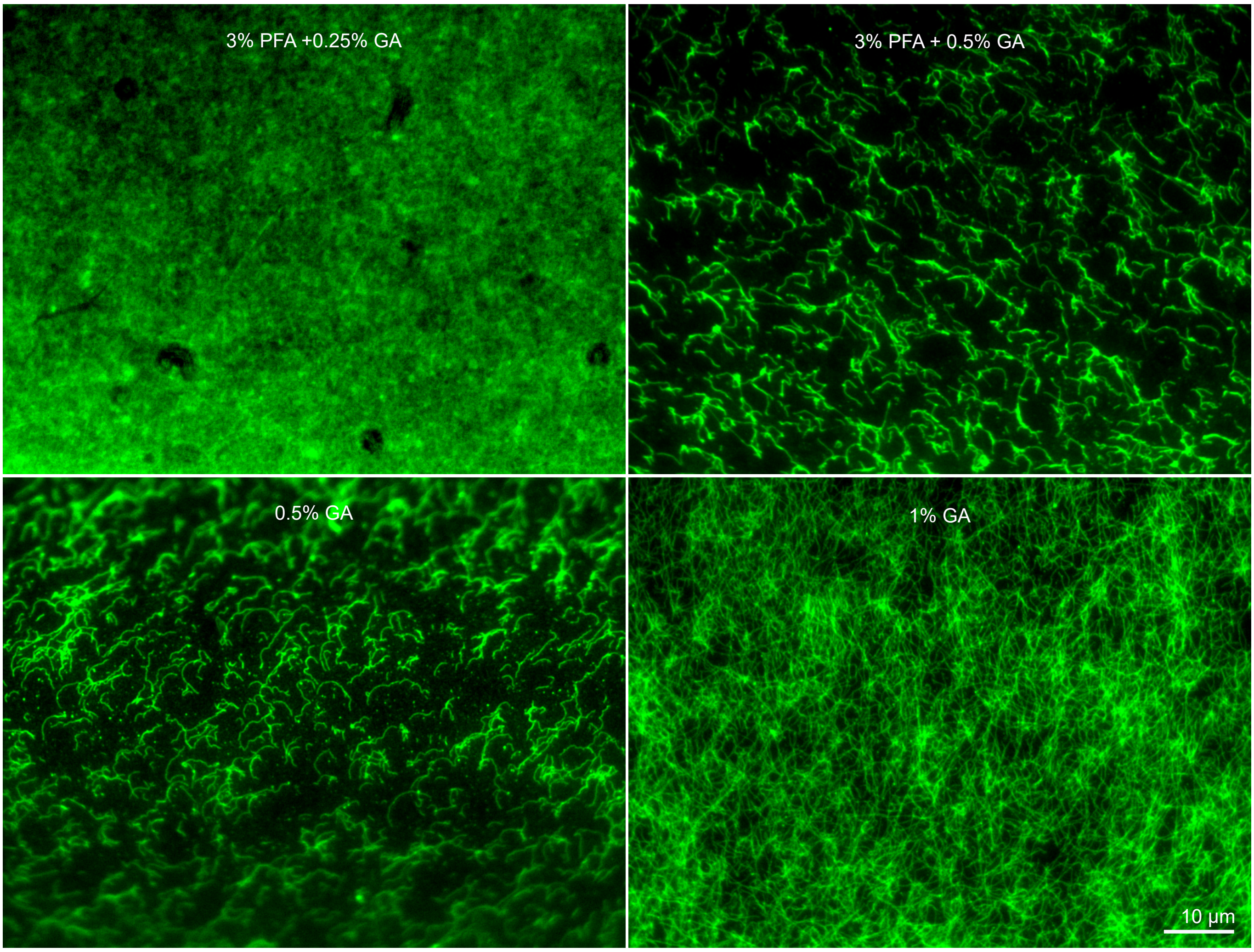

Supplementary Fig. 6. GA fixation of microtubules. This type of fixation provided optimal preservation of microtubule structure. However, it interferes with anchoring, preventing the implementation of ONE protocols. Microtubules are synthesized *in vitro*, stabilized with taxol (labeled with atto488), fixed with a mixture of PFA and GA or GA alone, attached as a monolayer onto the coverslip, and imaged using the fluorescence of atto488 under an Olympus IX71 microscope. (Left, top) Fixed with 3% PFA+0.25% GA, all the microtubules are disassembled, presumably into tubulin dimers. (Right, top) Fixed with 3% PFA+0.5% GA, mostly shorter filaments were observed, long filaments were broken. (Left, bottom) Fixed with 0.5% GA, results are very similar to the previous condition, suggesting that 3% PFA hardly played any role in microtubule fixation. (Right, bottom) Fixed with 1% GA, optimal fixation of microtubules is obtained, which preserved their morphology, evident from the presence of very long tubules.

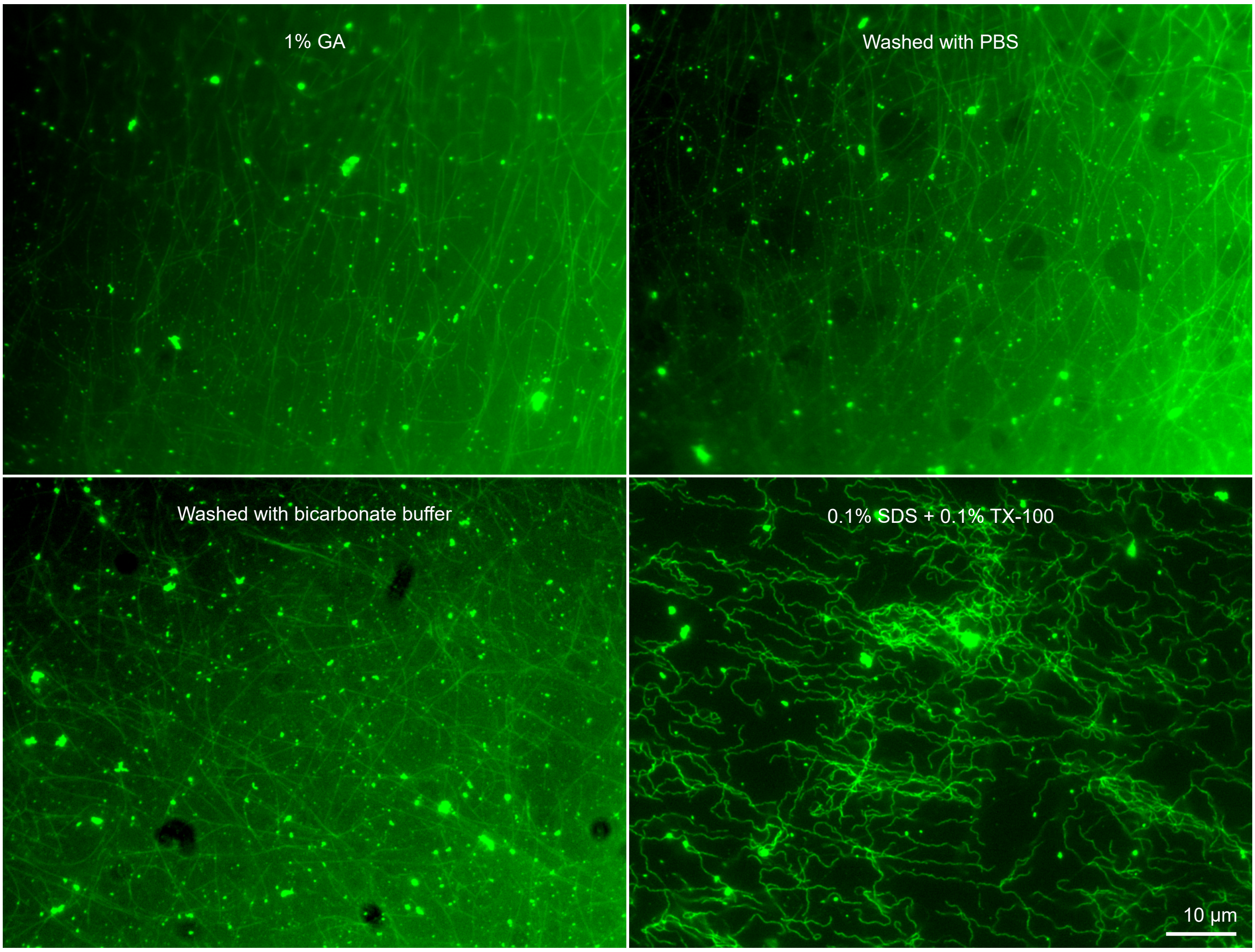

Supplementary Fig. 7. Attachment of microtubule onto the coverslip. *In vitro* synthesized microtubules were stabilized with taxol (labelled with Atto488), fixed with 1% GA, attached as a monolayer onto the coverslip following a method described before, washed with different buffers, and imaged using the fluorescence of Atto488, under an Olympus IX71 microscope. (Left, top) Microtubules fixated with 1% GA without any wash. (Right, top) Washed 3x with PBS buffer (pH 7.4). (Left, bottom) Washed 3x with bicarbonate buffer (pH 8.5). (Right, bottom) Washed 3x with 0.1% SDS+0.1% TX-100 in PBS (pH 7.4). This condition showed some distortion in the morphology of microtubules, as the filaments became curly, although they remained firmly attached with the glass surface of coverslip.

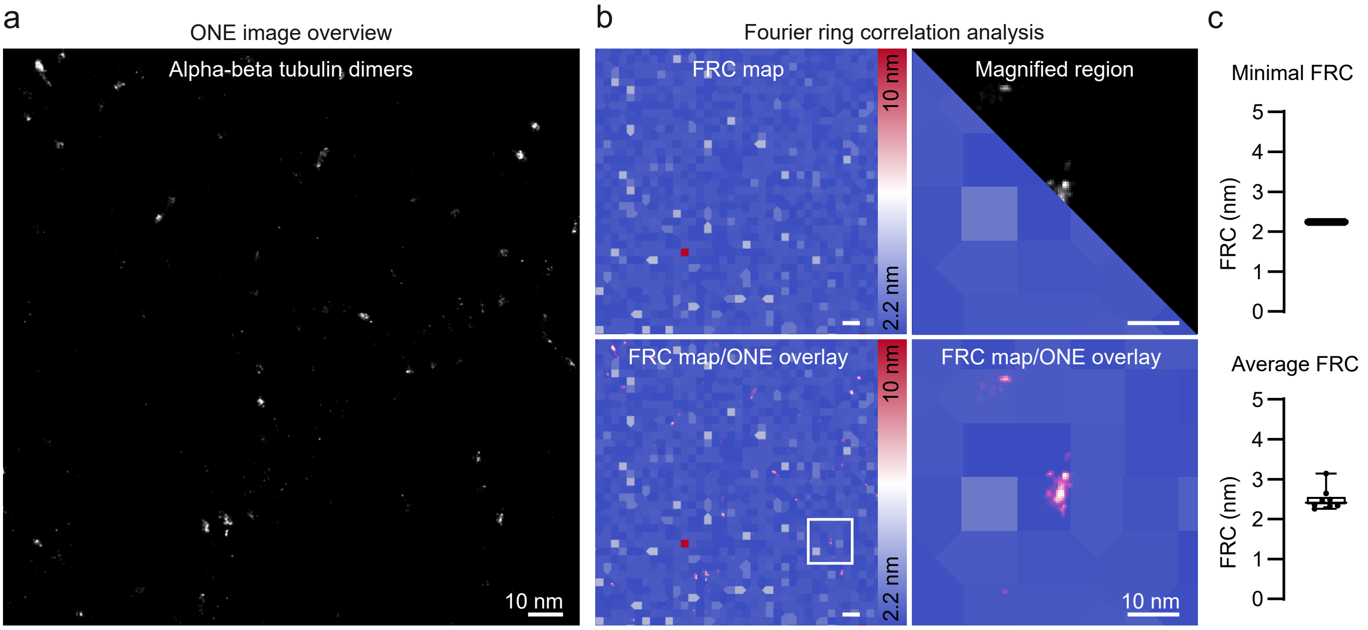

Supplementary Fig. 8. Fourier ring correlation analysis for tubulin dimers. a, Alpha-beta tubulin dimers, in a ONE overview image. **b**, Fourier ring correlation analysis. The first-left panel displays an exemplary FRC of ONE tubulin image with a pixel size of 98 nm. The bottom-left panel shows ONE image overlaid over FRC map using screen-blend mode. A magnified region featuring individual tubulin molecule represented in split and screen-blended modes. **c**, The graphs show box plots with the medians and the 25th percentile and the range values of Minimal and average calculated FRC, N = 8 images from 2 independent experiments.

Supplementary References

1 Gambarotto, D. et al. Imaging cellular ultrastructures using expansion microscopy (U-ExM). Nat Methods 16, 71-74 (2019). https://doi.org:10.1038/s41592-018-0238-1

2 Aktalay, A., Khan, T. A., Bossi, M. L., Belov, V. N. & Hell, S. W. Photoactivatable Carbo- and Silicon-Rhodamines and Their Application in MINFLUX Nanoscopy. Angew Chem Int Ed Engl 62, e202302781 (2023). https://doi.org:10.1002/anie.202302781

3 Descloux, A., Grussmayer, K.S. & Radenovic, A. Parameter-free image resolution estimation based on decorrelation analysis. Nat Methods 16, 918-924 (2019).
