# Supplementary material for: Microtubules as a versatile reference standard for expansion microscopy
Source: Commun Biol. 2025 Mar 26;8:499. doi: 10.1038/s42003-025-07967-3 (PMC11947214; doi:10.1038/s42003-025-07967-3)
Supplement: Supplementary file 5 — Reporting summary [file 42003_2025_7967_MOESM5_ESM.pdf]

nature portfolio

Corresponding author(s): Ali Shaib, Sarah Köster and Silvio Rizzoli

Last updated by author(s): Mar 18, 2025

Reporting Summary

Nature Portfolio wishes to improve the reproducibility of the work that we publish. This form provides structure for consistency and transparency in reporting. For further information on Nature Portfolio policies, see our Editorial Policies and the Editorial Policy Checklist.

Statistics

For all statistical analyses, confirm that the following items are present in the figure legend, table legend, main text, or Methods section.

n/a	Confirmed	
☐	☒	The exact sample size (*n*) for each experimental group/condition, given as a discrete number and unit of measurement
☐	☒	A statement on whether measurements were taken from distinct samples or whether the same sample was measured repeatedly
☐	☒	The statistical test(s) used AND whether they are one- or two-sided<br>*Only common tests should be described solely by name; describe more complex techniques in the Methods section.*
☐	☒	A description of all covariates tested
☐	☒	A description of any assumptions or corrections, such as tests of normality and adjustment for multiple comparisons
☐	☒	A full description of the statistical parameters including central tendency (e.g. means) or other basic estimates (e.g. regression coefficient) AND variation (e.g. standard deviation) or associated estimates of uncertainty (e.g. confidence intervals)
☐	☒	For null hypothesis testing, the test statistic (e.g. *F*, *t*, *r*) with confidence intervals, effect sizes, degrees of freedom and *P* value noted<br>*Give P values as exact values whenever suitable.*
☒	☐	For Bayesian analysis, information on the choice of priors and Markov chain Monte Carlo settings
☒	☐	For hierarchical and complex designs, identification of the appropriate level for tests and full reporting of outcomes
☒	☐	Estimates of effect sizes (e.g. Cohen's *d*, Pearson's *r*), indicating how they were calculated

Our web collection on statistics for biologists contains articles on many of the points above.

Software and code

Policy information about availability of computer code

Data collection	Data was acquired using Leica Application Suite for SP5 and SP8, Abberior Instruments Imspector v16.3 and Olympus CellSens Dimension 2.3
Data analysis	Custom code written in Matlab2017b and 2019b, GraphPad Prism 9, SigmaPlot 10 and Excel 2022 .

For manuscripts utilizing custom algorithms or software that are central to the research but not yet described in published literature, software must be made available to editors and reviewers. We strongly encourage code deposition in a community repository (e.g. GitHub). See the Nature Portfolio guidelines for submitting code & software for further information.

Data

Policy information about availability of data

All manuscripts must include a data availability statement. This statement should provide the following information, where applicable:

- Accession codes, unique identifiers, or web links for publicly available datasets
- A description of any restrictions on data availability
- For clinical datasets or third party data, please ensure that the statement adheres to our policy

Image data are available from the corresponding authors on reasonable request.

Human research participants

Policy information about studies involving human research participants and Sex and Gender in Research.

Reporting on sex and gender	*Use the terms sex (biological attribute) and gender (shaped by social and cultural circumstances) carefully in order to avoid confusing both terms. Indicate if findings apply to only one sex or gender; describe whether sex and gender were considered in study design whether sex and/or gender was determined based on self-reporting or assigned and methods used. Provide in the source data disaggregated sex and gender data where this information has been collected, and consent has been obtained for sharing of individual-level data; provide overall numbers in this Reporting Summary. Please state if this information has not been collected. Report sex- and gender-based analyses where performed, justify reasons for lack of sex- and gender-based analysis.*
Population characteristics	*Describe the covariate-relevant population characteristics of the human research participants (e.g. age, genotypic information, past and current diagnosis and treatment categories). If you filled out the behavioural & social sciences study design questions and have nothing to add here, write "See above."*
Recruitment	*Describe how participants were recruited. Outline any potential self-selection bias or other biases that may be present and how these are likely to impact results.*
Ethics oversight	*Identify the organization(s) that approved the study protocol.*

Note that full information on the approval of the study protocol must also be provided in the manuscript.

Field-specific reporting

Please select the one below that is the best fit for your research. If you are not sure, read the appropriate sections before making your selection.

☒ Life sciences ☐ Behavioural & social sciences ☐ Ecological, evolutionary & environmental sciences

For a reference copy of the document with all sections, see nature.com/documents/nr-reporting-summary-flat.pdf

Life sciences study design

All studies must disclose on these points even when the disclosure is negative.

Sample size	The largest possible numbers of experiments were performed, taking into account the high number of different experimental settings, and is well within the range of typical imaging experiments. No formal sample size calculation was performed.
Data exclusions	No experiments or data points were excluded (unless stated in figure legends).
Replication	In general 3 to 4 independent experiments were performed (experimental N is indicated for every dataset in the figure legends, with tens or hundreds of items (molecules, purified proteins, protein assemblies, synapses, synaptic structures, etc.) analyzed. All replications were successful. For expanded samples, each experimental N encompassed from 1 and up to 3 gel replicates.
Randomization	Not relevant for this manuscript.
Blinding	Most analyses relied on automated procedures which are not influenced by the nature of the sample. Blind analysis was applied when meaningful for the manual analysis and for the calmodulin experiment of which image acquisition was carried out blindly as well. Parkinson's disease data were imaged blindly and the analysis was performed by two independent investigators using two different image processing paradigms which was in agreement with automated script results using Matlab.

Reporting for specific materials, systems and methods

We require information from authors about some types of materials, experimental systems and methods used in many studies. Here, indicate whether each material, system or method listed is relevant to your study. If you are not sure if a list item applies to your research, read the appropriate section before selecting a response.

Materials & experimental systems

n/a	Involved in the study
☐	☒ Antibodies
☐	☒ Eukaryotic cell lines
☒	☐ Palaeontology and archaeology
☒	☐ Animals and other organisms
☒	☐ Clinical data
☒	☐ Dual use research of concern

Methods

n/a	Involved in the study
☒	☐ ChIP-seq
☒	☐ Flow cytometry
☒	☐ MRI-based neuroimaging

Antibodies

Antibodies used

Please refer to the relevant part of the Methods, describing at length all antibodies and nanobodies.

Validation

Rabbit alpha-Tubulin Abcam, Cat#ab18251, Cambridgshire, UK; validation in 286 citations, Berbari et al., 2012 - Cytoskeleton.
Mouse alpha-Tubulin Sigma Cat#T6199; Sigma-Aldrich, Darmstadt, Germany; validated in 105 citations, recent validation: Latremoliere et al., 2018 - Cell Rep.
Rabbit alpha Tubulin Synaptic Systems Cat#302 203, Göttingen, Germany; 4 citations, Wilhelm et al., 2014 - Science.
Mouse alpha Tubulin Synaptic Systems Cat#302 211; Göttingen, Germany; 15 citations, Groffen et al., 2010 - Science.
Rabbit NUP98-NUP96 Polyclonal antibody; Proteintech, Planegg-Martinsried, Germany, 6 citations - Neuron.
Rabbit NUP205 Polyclonal antibody; Proteintech, Planegg-Martinsried, Germany, 19 citations - Nat Met.

Eukaryotic cell lines

Policy information about cell lines and Sex and Gender in Research

Cell line source(s)

U2OS cell line from Cell Lines Service (CLS), Eppelheim, Germany.

Authentication

STR analysis according to the global standard ANSI/ATCC ASN-0002.1-2021 (2021) resulted in an authentic STR profile of the reference STR database - confirmed by the company.

Mycoplasma contamination

Negative in PCR assay - confirmed by the company.

Commonly misidentified lines
(See ICLAC register)

Not listed in the ICLAC database.
